# Vitamin D and diabetes in Koreans: analyses based on the Fourth Korea National Health and Nutrition Examination Survey (KNHANES), 2008–2009

**DOI:** 10.1111/j.1464-5491.2012.03575.x

**Published:** 2012-08

**Authors:** S Y Rhee, Y-C Hwang, H Y Chung, J-T Woo

**Affiliations:** Department of Endocrinology and Metabolism, Kyung Hee University School of Medicine and Research Institute of Endocrinology, Kyung Hee UniversitySeoul, Korea

**Keywords:** hyperglycaemia, insulin resistance, Korea, Type 2 diabetes mellitus, vitamin D deficiency

## Abstract

**Aims:**

A causal relationship between vitamin D deficiency and the incidence of diabetes mellitus has been suggested, but little research has been conducted on the Korean population.

**Methods:**

We analysed the glucose tolerance status and serum 25-hydroxyvitamin D concentrations in 12 263 subjects > 19 years old who were registered for the Korea National Health and Nutrition Examination Survey, 2008–2009.

**Results:**

Various demographic variables such as gender, age, season, resident area, physical activity, smoking, alcohol, marital status, education and occupation were associated with serum 25-hydroxyvitamin D concentrations. After adjusting for these variables as confounders, 25-hydroxyvitamin D concentrations in subjects with diabetes were significantly lower than those in subjects with normal glucose tolerance and those with impaired fasting glucose (*P* = 0.005). Compared with the ≥ 75 nmol/l subgroup of serum 25-hydroxyvitamin D concentration, the odds ratios and 95% confidence intervals for diabetes mellitus were 1.206 (95% CI 0.948–1.534) in the 50- to 74-nmol/l subgroup, 1.339 (1.051–1.707) in the 25- to 49-nmol/l subgroup and 1.759 (1.267–2.443) in the < 25-nmol/l subgroup. Compared with the serum ≥ 75-nmol/l 25-hydroxyvitamin D subgroup, serum insulin and homeostasis model assessment 2%B, a marker of insulin secretory capacity, were significantly higher, and homeostasis model assessment 2%S, a marker of insulin sensitivity, was significantly lower in the < 25- and 25- to 49-nmol/l serum 25-hydroxyvitamin D subgroups than those in the other subgroups (*P* < 0.001).

**Conclusions:**

The findings suggest that vitamin D deficiency, possibly involving altered insulin sensitivity, is associated with an increased risk for diabetes mellitus in the Korean population.

## Introduction

Vitamin D status is known to affect glucose metabolism [1,2]. Early studies on the role of vitamin D in glucose metabolism of humans were conducted on patients with Type 1 diabetes mellitus and found a significant correlation between vitamin D insufficiency and the risk of Type 1 diabetes [3]. In addition, artificial vitamin D supplements during early childhood were confirmed to play a role in the prevention of Type 1 diabetes [4,5].

Vitamin D insufficiency is also considered to influence the pathophysiology of Type 2 diabetes [6]. According to studies performed with various ethnic groups, a relatively consistent correlation is found between the prevalence of vitamin D insufficiency and Type 2 diabetes and the metabolic syndrome [7]. Moreover, vitamin D supplements improve insulin secretion and glucose tolerance in patients with Type 2 diabetes [8]. However, large-scale population-based studies examining the correlation between vitamin D insufficiency and Type 2 diabetes show heterogeneous results by ethnicity. Thus, the role of vitamin D in the pathophysiology of Type 2 diabetes has not been determined [7,9].

Over the past few decades, the prevalence of pre-diabetes and diabetes has rapidly increased in many Asian countries, including Korea [10]. Furthermore, Asian countries including Korea have a relatively high prevalence of vitamin D insufficiency compared with other regions around the world and compared with other ethnic groups [11,12]. These results suggest a causal relationship between increased prevalence of abnormal glucose tolerance and vitamin D insufficiency in Koreans. However, population-based studies to investigate this correlation in Koreans have rarely been conducted, and few studies have considered the correlations among vitamin D status, insulin secretion and insulin sensitivity in Koreans. Therefore, we conducted this study to determine the relationship between vitamin D insufficiency and glucose tolerance status and its mechanism based on nationally representative Korean population data from the Korea National Health and Nutrition Examination Survey (KNHANES IV) conducted in 2008–2009.

## Subjects and methods

### KNHANES IV

The Korea National Health and Nutrition Examination Survey is a nationwide, population-based, cross-sectional health survey. After the first KNHANES was performed in 1998, the second, third and fourth surveys were conducted in 2001, 2005 and 2007–2009, respectively.

KNHANES IV was conducted from July 2007 to December 2009. The subject population was all households and all those recorded on the 2005 Population and Housing Census in Korea. Relevant households were randomly selected through stratified and multistage probability sampling. As rolling survey methods were used for sampling, the sample for each year was a probability sample representing all parts of the country, and each rolling sample had homogenous and independent characteristics compared with the others.

KNHANES IV was performed with all members of sampled households aged > 1 year and consisted of a health interview, a health examination and a nutrition survey. The health interview and health examination were conducted in adjacent public offices or in equipped mobile examination centres, whereas the nutrition survey was conducted through house-to-house enquiries. A total of 31 705 KNHANES IV sampled subjects in 9421 households were screened. Among them, 23 632 (74.5%) participated in the health interview and health examination survey and 22 137 (81.8%) finished the nutrition survey. All participants in this survey signed informed consent forms.

### Study subjects

This study utilized the 2008 and 2009 KNHANES IV data, which included sampling 25-hydroxyvitamin D (25OHD) concentrations in subjects. Of the subjects aged > 19 years who participated in KNHANES IV from February 2008 to December 2009 (*n* = 13 720), demographic characteristics such as physical activity (*n* = 13 610), smoking (*n* = 13 621), alcohol use (*n* = 13 631), marital status (*n* = 13 688), education (*n* = 13 623) and occupation (*n* = 13 581) were verified through the health interview, and subjects in whom serum 25OHD (*n* = 13 166), fasting plasma glucose (*n* = 13 132), fasting serum insulin (*n* = 13 166) and glucose tolerance status (*n* = 12 669) were investigated were selected as eligible subjects. After excluding patients with active chronic diseases such as active tuberculosis (*n* = 28), chronic kidney disease (*n* = 36), chronic liver disease (*n* = 99) and hepatocellular carcinoma (*n* = 13) to eliminate plausible factors affecting vitamin D metabolism of the subjects, 12 263 were screened as final enrolled subjects.

### Study methods

We investigated the difference in serum 25OHD levels by anthropometric and demographic characteristics and glucose tolerance status based on the health interview and health examination data of KNHANES IV. Additionally, the difference in the risk of diabetes based on the 25OHD level was also examined by subdividing the subjects by 25OHD concentration. Finally, the difference in insulin sensitivity and insulin secretion capacity was evaluated based on the serum 25OHD concentration.

Blood samples of all subjects were collected after > 8 h of fasting. Specimens were immediately centrifuged, aliquoted, frozen at −70 °C and moved to the central laboratory (NeoDIN Medical Institute, Seoul, South Korea), where they were analysed within 24 h. Fasting plasma glucose concentrations were measured using an automated analyser with an enzymatic assay (Pureauto S GLU: Daiichi, Tokyo, Japan). Serum insulin concentrations were measured using a gamma counter with an immunoradiometric assay (INS-Irma; Biosource, Nivelles, Belgium). Serum 25OHD concentrations were measured using a gamma counter with a radioimmunoassay (25-Hydroxyvitamin D ^125^I RIA Kit; DiaSorin, Still Water, MN, USA). The interassay coefficients of variations for the serum 25OHD assay were 5.0–7.6% in KNHANES 2008 samples and 2.8–6.2% in KNHANES 2009 samples.

To determine differences based on the demographic characteristics and 25OHD level, the subjects were subdivided according to the characteristics of each variable. BMI was classified into (1) < 18.5 kg/m^2^, (2) 18.5–22.9 kg/m^2^, (3) 23–24.9 kg/m^2^ and (4) ≥ 25 kg/m^2^, based on the diagnostic criteria of the Korean Society for the Study of Obesity [13]. To investigate the difference based on examination season, the time of the health examination was divided into four subgroups: (1) January–March, (2) April–June, (3) July–September and (4) October–December. Sixteen resident areas of KNHANES were classified into (1) urban areas, including metropolitan cities such as Seoul, the capital city, Busan, Daegu, Incheon, Gwangju, Daejeon and Ulsan, as well as metropolitan areas such as Gyeonggi province and (2) rural areas, comprising Chungbuk, Chungnam, Gangwon, Gyeongbuk, Gyeongnam, Jeonnam, Jeonbuk and Jeju province. The physical activity of the subjects was categorized by investigating recreational physical activity for the previous 1 week into: (1) none, no activity; (2) mild, > 30 min of walking more than 5 days per week; (3) moderate, > 30 min of moderate physical activity in which the subject was tired compared with an ordinary level or breathing slightly hard more than 5 days per week; and (4) vigorous physical activity, > 20 min of vigorous physical activity in which the subject was exhausted compared with an ordinary level or breathing hard more than 3 days per week. Current smokers were defined as those who had smoked more than five packs of cigarettes during their life and were smoking currently, and non-smokers were all others. Regular alcohol drinkers were those who drank alcohol currently more than one time per month, and non-drinkers were all others. Marital status was classified as (1) unmarried, (2) married/living together and (3) separated/divorced/bereaved. Education was categorized as (1) graduation from elementary school or lower, (2) graduation from middle school, (3) graduation from high school and (4) graduation from college or higher. Occupation was divided into seven groups: (1) managers, professionals, technicians and associated professionals; (2) clerical support workers; (3) service and sales workers; (4) skilled agricultural, forestry and fishery workers; (5) craft and related trades workers, plant and machine operators and assemblers; (6) elementary occupations; and (7) housewife, student and unemployed, based on the 6th Korean Standard Classification of Occupations from the Korean National Statistical Office created by following the International Standard Classification of Occupations of the International Labour Organization [14].

The insulin sensitivity and insulin secretion capacity of the subjects were evaluated based on the results of the health examination, glucose tolerance status and 25OHD status. Glucose tolerance status was divided into normal glucose tolerance, impaired fasting glucose and diabetes based on the fasting plasma glucose concentration and the diagnostic criteria of the American Diabetes Association [15]. If subjects were confirmed to have diabetes by physicians or had used oral hypoglycaemic agents or insulin injections, they were classified as having diabetes regardless of fasting glucose level. The 25OHD status of subjects was divided into four groups: ≥ 75 nmol/l, 50–74 nmol/l, 25–49 nmol/l and < 25 nmol/l, based on the vitamin D insufficiency criterion widely accepted by professionals [2,16]. The insulin sensitivity and insulin secretion capacity of the subjects were assessed with fasting plasma glucose and fasting serum insulin. Homeostasis model assessment (HOMA) 2%S and HOMA2%B were utilized, employing the HOMA2 method, a computerized improvement on the standard HOMA method [17,18]. The HOMA2 method reflects insulin secretion capacity and insulin resistance more accurately than did the previous HOMA method [17,18]. Furthermore, a disposition index (DI) to adjust insulin resistance factors affecting insulin secretion, HOMA2-DI, was defined as the value obtained by multiplying HOMA2%S by HOMA2%B [19]. To exclude the effect of medications, subjects who had taken oral hypoglycaemic agents or who had a history of insulin treatment (*n* = 730) were not analysed with the HOMA2 method.

### Statistical analysis

All data are presented as the means ± se or as proportions. Predictive Analystics SoftWare (PASW; version 18.0) (SPSS Inc., Chicago, IL, USA) was utilized for the statistical analysis and data management. The difference in serum 25OHD concentrations based on the subject characteristics and on glucose tolerance subgroups was determined, and the influence of other variables was adjusted using an analysis of covariance (ANCOVA). To investigate the degree of diabetes risk based on the serum 25OHD concentrations, odds ratios for the prevalence of diabetes were calculated using the ≥ 75 nmol/l 25OHD subgroup as the standard, and the effect of confounders was adjusted using logistic regression analysis. The effect of confounders was adjusted using an ANCOVA to compare the significance of insulin secretion and insulin sensitivity among serum 25OHD subgroups. *P*-values < 0.05 were considered significant.

## Results

### 25OHD status

The serum 25OHD levels of the subjects ranged from 4.94 to 167.13 nmol/l (median 44.55 nmol/l). Subjects with 25OHD levels ≥ 75, 50–74, 25–49 and < 25 nmol/l accounted for 7.8% (*n* = 962), 30.3% (*n* = 3714), 54.4% (*n* = 6668) and 7.5% (*n* = 919) of the subjects, respectively.

### Differences in serum 25OHD by demographic characteristics

**FIGURE 1:**
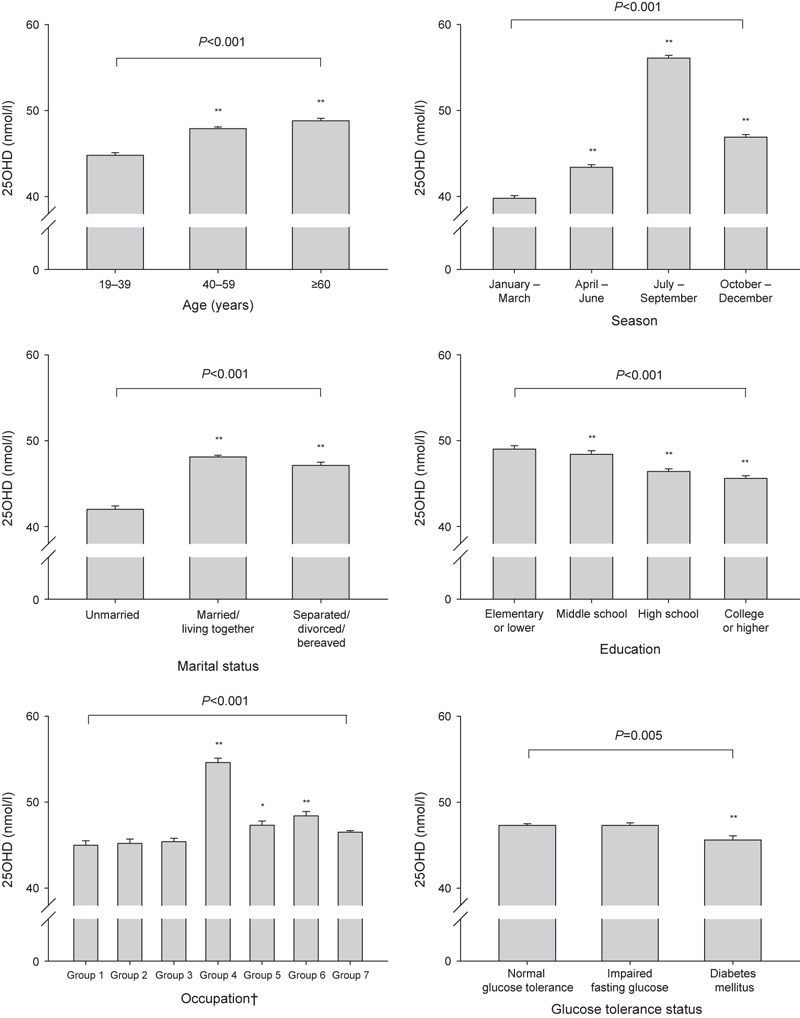
Serum 25-hydroxyvitamin D (25OHD) concentrations by demographic characteristics and glucose tolerance status adjusted for all other significant variables. Mean ± se. *P < 0.05, **P < 0.01 by post-hoc analysis. †Occupation group refers to the Korean Standard Classification of Occupations-6 classification: group 1: managers, professionals, technicians and associate professionals; group 2, clerical support workers; group 3, service and sales workers; group 4, skilled agricultural, forestry and fishery workers; group 5, craft and related trades workers, plant and machine operators, and assemblers; group 6, elementary occupations; group 7, housewife, student and unemployed.

The serum 25OHD concentrations of the subjects were significantly different based on the demographic characteristics (Fig. 1 and see also Supporting Information, Table S1). Male subjects represented 42.8% of all subjects and their serum 25OHD levels were significantly higher than those of females. Subjects aged 19–39, 40–59 and ≥ 60 years accounted for 33.8, 37.3 and 28.9%, respectively, and the older age group recorded significantly higher 25OHD concentrations compared with those in the younger age groups. The 25OHD concentration was also significantly different according to health examination season; the 25OHD concentration was lowest among the subjects who underwent the examination during January–March. The 25OHD concentration was significantly higher in rural areas than that in urban areas. Subjects who performed no regular exercise and those who performed mild, moderate and vigorous physical activity included 42.9, 30.2, 9.9 and 16.9% of all subjects, respectively, and serum 25OHD level increased significantly with higher intensity physical activity. The 25OHD level of current smokers was lower than that of non-smokers, whereas the 25OHD level of regular drinkers was significantly higher than that of non-drinkers. Serum 25OHD concentration also differed by marital status and was significantly higher among subjects in the married/living together and separated/divorced/bereaved groups than among unmarried subjects. A difference in 25OHD concentrations based on education status was also observed, and the concentration of subjects who had graduated from high school or higher was significantly lower than that of subjects who had completed elementary school or less. The difference in 25OHD concentrations by occupation was also significant. The concentration in skilled agricultural, forestry and fishery workers was significantly higher than that among subjects in other occupations, with the exception of manual workers. No difference in 25OHD concentration based on BMI was observed.

### Difference in serum 25OHD based on glucose tolerance

When the subjects were classified by glucose tolerance, 9.6, 18.9 and 71.5% were diagnosed with diabetes, impaired fasting glucose and normal glucose tolerance, respectively (Fig. 1). The difference between normal glucose tolerance and impaired fasting glucose was not significant, but the 25OHD level of subjects with diabetes was significantly lower than that of subjects with normal glucose tolerance and impaired fasting glucose. Even after oral hypoglycaemic agent or insulin users were excluded from the patients with diabetes, the same analysis found no change in the significance of the results (data not shown).

### Difference in diabetes risk based on serum 25OHD concentration

When the difference in diabetes risk based on the 25OHD concentration was investigated, the odds ratios (OR) for the 50- to < 75-nmol/l subgroup compared with the ≥ 75-nmol/L 25OHD subgroup was not significant [OR 1.206; 95% confidence interval (CI), 0.948–1.534]. However, the odds ratios for the 25- to < 50-nmol/l subgroup and the < 25-nmol/l subgroup were 1.339 (95% CI 1.051–1.707) and 1.759 (95% CI 1.267–2.443), respectively, indicating that the risk of diabetes was significantly greater in these groups (Table 1).

**Table 1:** Diabetes odds ratios according to 25-hydroxyvitamin D (25OHD) subgroup

	Diabetes mellitus			
			
25OHD subgroup	Yes	No	OR (95% CI)	*P*
≥ 75 nmol/l	101	861	-	
50 to < 75 nmol/l	384	3330	1.206 (0.948–1.534)	0.127
25 to < 50 nmol/l	600	6068	1.339 (1.051–1.707)	0.018
< 25 nmol/l	96	823	1.759 (1.267–2.443)	0.001

Adjusted for sex, age, season, location, physical activity, smoking, alcohol, marital status, education and occupation.

### Difference in insulin secretion and insulin sensitivity based on serum 25OHD concentration

The difference in fasting plasma glucose, fasting serum insulin, HOMA2%S and HOMA2%B based on the 25OHD level was determined (Table 2). Fasting plasma glucose was not significantly different based on the 25OHD levels, but fasting serum insulin, HOMA2%B and HOMA2%S showed significant differences based on the serum 25OHD level. While fasting serum insulin and HOMA2%B in the 25- to < 50-nmol/l and < 25-nmol/l 25OHD subgroups tended to increase gradually compared with the ≥ 75 nmol/l 25OHD subgroup, the HOMA2%S values for the first two groups tended to decrease steadily. According to a post-hoc analysis, the difference was significant for the < 50-nmol/l 25OHD subgroup. After adjusting for the effect of insulin resistance on insulin secretion, the difference in HOMA2 disposition index based on 25OHD concentration was not significant. Even after subjects with a history of oral hypoglycaemic agent or insulin use were included in the analysis, no changes in significance were observed (data not shown).

**Table 2:** Comparisons of fasting plasma glucose, fasting serum insulin, HOMA2%S, and HOMA2%B according to serum 25OHD subgroup in subjects with normal glucose tolerance, impaired fasting glucose and in drug-naive subjects with diabetes

	Serum 25OHD concentration (nmol/l)				
		
Variables	< 25	25 to < 50	50 to < 75	≥ 75	*P*
Glucose (mmol/l)	5.24 ± 0.03	5.27 ± 0.01	5.26 ± 0.02	5.25 ± 0.03	0.656
Insulin (pmol/l)	65.5 ± 1.1*	67.1 ± 0.4†	63.8 ± 0.5	61.2 ± 1.1	< 0.001
HOMA2%B (%)	101.3 ± 1.0†	101.7 ± 0.4†	98.5 ± 0.5	96.1 ± 1.0	< 0.001
HOMA2%S (%)	94.8 ± 1.2	92.9 ± 0.4^b^	96.1 ± 0.6	98.6 ± 1.2	< 0.001
HOMA2 disposition index	8983.0±89.6	8788.4 ± 32.9	8886.3 ± 44.5	8897.2 ± 88.0	0.084

Mean ± se.

* *P* < 0.05,

† *P* < 0.01 by post-hoc analysis, 25OHD ≥ 75-nmol/l subgroup as reference.

All variables adjusted for sex, age, season, location, physical activity, smoking, alcohol, marital status, education and occupation.

25OHD, 25-hydroxyvitamin D; %B, beta cell function; HOMA, homeostasis model assessment; %S, insulin sensitivity.

## Discussion

Diverse demographic characteristics including age, gender, examination season, resident area, physical activity, smoking, alcohol use, marital status, education and occupation were found to influence vitamin D status in Korean adults. Some of these results were in agreement with the results of previous studies; however, others were novel findings in Koreans compared with previous studies.

The prevalence of vitamin D insufficiency was very high in > 60% of Korean adults and the average 25OHD concentration of the younger age subgroup was significantly lower than that of the older age group. Similar results were reported in another study [20]. Although this finding cannot be fully explained, it was suggested that the younger age subgroup had a relatively high rate of jobs with less sun exposure, such as office work, whereas the older age group had a higher rate of occupations with significant physical activity [20]. The National Diet and Nutrition Survey 1992–2001 in England also revealed a higher prevalence of vitamin D insufficiency in a younger age group [21]. Second, no significant difference was noted in serum 25OHD levels based on the BMI in Koreans. Because vitamin D generally shows a high affinity for fat tissue, obesity decreases serum 25OHD concentrations through reduced bioavailability [2,16]. However, this tendency was not observed in this study, perhaps because the average BMI of Koreans is relatively lower compared with that of other races and because the rate of severe obesity provoking a significant influence on vitamin D bioavailability was not high. Third, mean 25OHD concentrations were significantly higher among those who drank alcohol regularly. Previously conducted case–control studies reported that the mean 25OHD level in alcohol abusers was significantly lower than that in healthy control subjects [22]. However, regular drinking in the present study was defined as socially acceptable alcohol consumption, and this behaviour could be related to greater social activity, which may have positively affected serum 25OHD concentrations. Fourth, marital status also affected serum 25OHD concentrations in this study, suggesting that marital status can affect important lifestyle and nutritional factors. Finally, the difference in 25OHD level between subjects with normal glucose tolerance and impaired fasting glucose was not significant; however, 25OHD in subjects with diabetes was significantly lower than that in other subgroups, suggesting that 25OHD may play a preventive role against deterioration in glucose tolerance. Results from a 3-year double-blind, randomized, controlled trial on 314 Caucasian adults indicated that calcium and vitamin D supplementation of subjects with impaired fasting glucose may attenuate increases in glycaemia and insulin resistance [23]; however, these effects have not been validated.

Serum 25OHD levels in subjects with diabetes were significantly lower than those in subjects with normal glucose tolerance, and the odds ratios for diabetes increased significantly in < 50-nmol/l 25OHD subgroups. This finding suggests that vitamin D insufficiency is related to diabetes risk in Koreans and that vitamin D status may affect glucose metabolism as well as bone metabolism in Koreans with vitamin D insufficiency.

The observation that vitamin D has a positive effect on glucose metabolism by stimulating insulin secretion in β-cells and improving insulin sensitivity is well known through experimental models. Vitamin D receptors have been identified on β-cells and vitamin D boosts the conversion of proinsulin to insulin and augments insulin secretion [6]. Skeletal muscle is a major component of insulin sensitivity and also contains vitamin D receptors. A clamp study revealed that vitamin D supplementation improved insulin sensitivity in skeletal muscle [24]. However, not all experimental studies have found similar results. A study using transgenic vitamin D receptor (VDR) knockout mice reported that the correlation between vitamin D and glucose tolerance was heterogeneous, based on the genetic background of the strains used to make the transgenic mice [6].

Heterogeneity by genetic background has also been found in large-scale human studies. The NHANES III study in the USA found an inverse correlation between vitamin D status and diabetes risk or insulin resistance in non-Hispanic white and Mexican Americans [9]. However, the relationship was not found in a non-Hispanic black subgroup [9]. A study conducted with 5677 New Zealand Polynesians and Caucasians showed that the 25OHD3 levels of the Maori and Pacific Islanders were significantly lower than that of Caucasians and the difference partially explained the higher prevalence of impaired glucose tolerance and diabetes in Polynesians than in Caucasians [25]. These population-based study findings indicate that hypovitaminosis D might be a significant risk factor for diabetes in some ethnic groups, but the effect could be relatively low in other groups with different genetic backgrounds; therefore, similar studies with various ethnic groups including Koreans should be performed. Although objective bases to explain these ethnic differences are still insufficient, some investigators insist that the differences are related to a difference in the threshold of physiological action of hormones affecting vitamin D metabolism [9].

We indirectly estimated how vitamin D status could affect the pathophysiology of diabetes in Koreans. The mean HOMA2%S of the subjects was significantly lower and fasting serum insulin and HOMA2%B were significantly higher in the < 50-nmol/l 25OHD subgroups, but no difference in HOMA2 disposition index was observed between each 25OHD subgroup. This result indicates that the diabetes risk attributable to vitamin D insufficiency is mainly related to decreased insulin sensitivity rather than β-cell dysfunction in Koreans; thus, higher fasting serum insulin levels and HOMA2%B in the < 50-nmol/l 25OHD subgroups may reflect compensatory insulin secretion to overcome insulin resistance. However, the variance in HOMA2%B and HOMA2%S based on serum 25OHD status was < 10%, so it appears that 25OHD does not play an absolute role in glucose metabolism in Koreans. Actually, no significant differences in fasting blood glucose were observed based on the 25OHD concentrations in the current study.

Another remarkable finding in this study was that the 25OHD cut-off for deterioration in glucose metabolism was identical to that for bone metabolism. Although supporting evidence is insufficient, this finding suggests that the physiological roles of vitamin D in glucose and in bone metabolism *in vivo* may be closely related. However, further studies are needed to identify the mechanism of such correlations.

This study had some limitations. First, as KNHANES IV was a cross-sectional study, the causal relationship between vitamin D status and glucose tolerance status was unclear. Additionally, the HOMA2 method used in this study is not a gold standard to measure insulin secretion and insulin sensitivity. Despite these limitations, the results are meaningful because they revealed a significant correlation between vitamin D insufficiency and diabetes risk and clarified important characteristics of vitamin D status in Koreans compared with other races using a large number of subjects representing the entire Korean population. Additionally, we estimated the putative involvement of 25OHD in glucose metabolism in the Korean population by means of HOMA2, which has been one of the widely accepted methods in epidemiological studies.

In conclusion, diverse demographic characteristics influenced vitamin D status in Korean adults. Additionally, low 25OHD concentration was significantly related to diabetes risk and the effect of 25OHD on glucose metabolism was more closely associated with decreased insulin sensitivity in the Korean population. Future large-scale longitudinal studies that overcome the current study’s limitations will present a more detailed view of the pathophysiological role of vitamin D in glucose metabolism.

## Abbreviations

25OHD: 25-hydroxyvitamin D
%B: beta cell function
HOMA: homeostasis model assessment
KNHANES: Korea National Health and Nutrition Examination Survey
%S: insulin sensitivity

## Supporting Information

**Table S1.** Serum 25OHD concentrations by demographic characteristics adjusted for all other significant variables.
