# Supplementary material for: Vitamin D and diabetes in Koreans: analyses based on the Fourth Korea National Health and Nutrition Examination Survey (KNHANES), 2008–2009
Source: Diabet Med. 2012 Aug;29(8):1003–10. doi: 10.1111/j.1464-5491.2012.03575.x (PMC3504347; doi:10.1111/j.1464-5491.2012.03575.x)
Supplement: Supplementary file 1 [file dme0029-1003-SD1.doc]

Supplement Table. Serum 25OHD concentrations by demographic characteristics adjusted for all other significant variables.

| Variables | n (%) | 25OHD (nmol/L) | *post-hoc* *p* | total *p* |
| --- | --- | --- | --- | --- |
| Sex | | | | |
| men | 5,244 (42.8) | 51.8±0.3 | - | <0.001 |
| women | 7,019 (57.2) | 43.6±0.2 | - |
| Age (years) | | | | |
| 19–39 | 4,141 (33.8) | 44.8±0.3 | referent | <0.001 |
| 40–59 | 4,577 (37.3) | 47.9±0.2 | <0.001 |
| ≥60 | 3,545 (28.9) | 48.8±0.3 | <0.001 |
| BMI (kg/m^2^) | | | | |
| < 18.5 | 571 (4.7) | 47.0±0.7 | referent | 0.949 |
| 18.5–22.9 | 4,899 (39.9) | 47.2±0.2 | 1.000 |
| 23–24.9 | 2,931 (23.9) | 47.3±0.3 | 1.000 |
| ≥ 25 | 3,862 (31.5) | 47.0±0.3 | 1.000 |
| Season | | | | |
| January–March | 2,329 (19.0) | 39.8±0.3 | referent | <0.001 |
| April–June | 3,235 (26.4) | 43.4±0.3 | <0.001 |
| July–September | 3,305 (27.0) | 56.1±0.3 | <0.001 |
| October–December | 3,394 (27.7) | 46.9±0.3 | <0.001 |
| Resident area | | | | |
| urban area | 7,716 (62.9) | 45.2±0.2 | - | <0.001 |
| rural area | 4,547 (37.1) | 50.3±0.2 | - |
| Physical activity | | | | |
| none | 5,264 (42.9) | 45.6±0.2 | referent | <0.001 |
| mild | 3,708 (30.2) | 47.5±0.3 | <0.001 |
| moderate | 1,216 (9.9) | 49.1±0.5 | <0.001 |
| vigorous | 2,075 (16.9) | 49.0±0.4 | <0.001 |
| Smoking | | | | |
| no smoker | 9,584 (78.2) | 47.5±0.2 | - | <0.001 |
| current smoker | 2,679 (21.8) | 45.6±0.4 | - |
| Alcohol | | | | |
| no drinker | 5,696 (46.4) | 45.8±0.2 | - | <0.001 |
| regular drinker | 6,567 (53.6) | 48.2±0.2 | - |
| Marital status | | | | |
| unmarried | 1,745 (14.2) | 42.0±0.4 | referent | <0.001 |
| married/living together | 8,833 (72.0) | 48.1±0.2 | <0.001 |
| separated/divorced/bereaved | 1,685 (13.7) | 47.1±0.4 | <0.001 |
| Education | | | | |
| graduation from elementary school or lower | 3,369 (27.5) | 49.0±0.4 | referent | <0.001 |
| graduation from middle school | 1,368 (11.2) | 48.4±0.4 | 1.000 |
| graduation from high school | 4,287 (35.0) | 46.4±0.3 | <0.001 |
| graduation from college or higher | 3,239 (26.4) | 45.6±0.3 | <0.001 |
| Occupation^a^ | | | | |
| group 1 | 1,403 (11.4) | 45.0±0.5 | referent | <0.001 |
| group 2 | 942 (7.7) | 45.2±0.5 | 1.000 |
| group 3 | 1,547 (12.6) | 45.4±0.4 | 1.000 |
| group 4 | 1,211 (9.9) | 54.6±0.5 | <0.001 |
| group 5 | 1,174 (9.6) | 47.3±0.5 | 0.010 |
| group 6 | 1,097 (8.9) | 48.4±0.5 | <0.001 |
| group 7 | 4,889 (39.9) | 46.5±0.2 | 0.123 |
| Glucose tolerance status | | | | |
| normal glucose tolerance | 8,766 (71.5) | 47.3±0.2 | referent | 0.005 |
| impaired fasting glucose | 2,316 (18.9) | 47.3±0.3 | 1.000 |
| diabetes mellitus | 1,181 (9.6) | 45.6±0.5 | 0.005 |
| Total | 12,263 (100.0) | - |  | - |

Mean ± S.E.

^a^ Occupation group referred to the KSCO-6 classification. Group 1 indicates managers, professionals, technicians and associate professionals; group 2, clerical support workers; group 3, service and sales workers; group 4, skilled agricultural, forestry and fishery workers; group 5, craft and related trades workers, plant and machine operators, and assemblers; group 6, elementary occupations; group 7, housewife, student, and unemployed.
